# 233. All *Clostridium butyricum* strains isolated from blood cultures were derived from probiotics: a single-centre retrospective study

**DOI:** 10.1093/ofid/ofad500.306

**Published:** 2023-11-27

**Authors:** Ryuichi M Sada, Daisuke Motooka, Satoshi Kutsuna, Shigeto Hamaguchi, Go Yamamoto, Akiko Ueda

**Affiliations:** Osaka University Graduate School of Medicine, Suita, Osaka, Japan; Research Institute for Microbial Diseases, Osaka University,, Suita, Osaka, Japan; Graduate School of Medicine/Faculty of Medicine, Osaka University, Suita, Osaka, Japan; Osaka university Graduate School of Medicine, Suita, Osaka, Japan; Osaka University Graduate School of Medicine, Suita, Osaka, Japan; Laboratory for Clinical Investigation, Osaka University Hospital, Suita, Osaka, Japan

## Abstract

**Background:**

*Clostridium butyricum*

(*C. butyricum*) is a strictly anaerobic, Gram-positive, spore-forming bacillus named for its capacity to produce high amounts of butyric acid. Some strains of *C. butyricum* involved in infectious diseases are known to be currently used as probiotics in Asia. Especially in Japan, MIYA-BM^®^ is one of the probiotics commonly used as prescription drugs and it contains *C. butyricum* MIYAIRI 588 strain (CBM588). On the other hand, other strains involved in infectious diseases are known to be pathogenic. Bacteremia due to *C. butyricum* is a rare condition, and the prevalence, clinical features, and bacteriological and genetic origins of the condition are unknown.

**Methods:**

We conducted a retrospective cohort study of medical records of patients to detect cases of *C. butyricum* bacteremia in Osaka University Hospital from September 19, 2011, to February 5, 2023. We analyzed the whole-genome sequencing of *C. butyricum* strains from positive blood culture samples, as well as strains from the probiotic MIYA-BM^®^. We also analyzed to determine the homology between these strains.

**Results:**

Out of a total of 6576 positive blood culture samples, we detected five cases (0.08%) of bacteremia due to *C. butyricum*. Whole-genome analysis showed that the genes of each strain had only 1-33 mutations compared to the genes of the CBM588 strain, identifying them as the same clone derived from MIYA-BM^®^. The detail of case characteristics is shown in Table 1. All patients had bacteremia during hospitalization, with two male and three female patients. Four patients had concurrent use of MIYA-BM^®^. Four patients had immunocompromised conditions, and two patients had end-stage kidney disease on dialysis. All patients had a fever and abdominal symptoms such as diarrhea and pain. A patient with non-occlusive mesenteric ischemia died within 90 days.

**Table.**
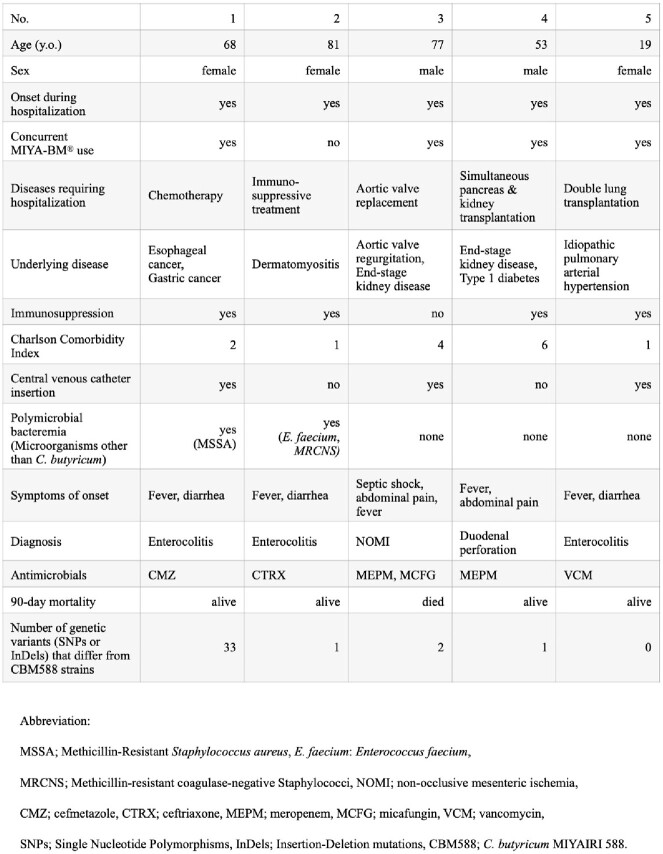
Detailed clinical and microbiological information on five cases.

**Conclusion:**

All *C. butyricum* strains detected from blood cultures in our hospital were derived from probiotics. Although *C. butyricum* bacteremia is rare condition, the use of MIYA-BM^®^ use in hospitalized patients with multiple medical interventions is considered to cause *C. butyricum* bacteremia.

**Disclosures:**

**All Authors**: No reported disclosures

